# Thermally-responsive Virus-like Particle for Targeted Delivery of Cancer Drug

**DOI:** 10.1038/s41598-019-40388-x

**Published:** 2019-03-08

**Authors:** Qiu Xian Thong, Roya Biabanikhankahdani, Kok Lian Ho, Noorjahan Banu Alitheen, Wen Siang Tan

**Affiliations:** 1 Department of Microbiology, Faculty of Biotechnology and Biomolecular Sciences, Universiti Putra Malaysia, 43400 UPM Serdang, Selangor Malaysia; 2 Department of Microbiology, College of Science, Agriculture and Modern Technologies, Shiraz Branch, Islamic Azad University, Shiraz, Iran; 3 Department of Pathology, Faculty of Medicine and Health Sciences, Universiti Putra Malaysia, 43400 UPM Serdang, Selangor Malaysia; 4 Department of Cell and Molecular Biology, Faculty of Biotechnology and Biomolecular Sciences, Universiti Putra Malaysia, 43400 UPM Serdang, Selangor Malaysia; 5 Institute of Bioscience, Universiti Putra Malaysia, 43400 UPM Serdang, Selangor Malaysia

## Abstract

Multifunctional nanocarriers displaying specific ligands and simultaneously response to stimuli offer great potentials for targeted and controlled drug delivery. Several synthetic thermally-responsive nanocarriers have been studied extensively for hyperthermia incorporated chemotherapy. However, no information is available on the application of virus-like particle (VLP) in thermally-controlled drug delivery systems. Here, we describe the development of a novel multifunctional nanovehicle based on the VLP of *Macrobrachium rosenbergii* nodavirus (MrNVLP). Folic acid (FA) was covalently conjugated to lysine residues located on the surface of MrNVLP, while doxorubicin (Dox) was loaded inside the VLP using an infusion method. This thermally-responsive nanovehicle, namely FA-MrNVLP-Dox, released Dox in a sustained manner and the rate of drug release increased in response to a hyperthermia temperature at 43 °C. The FA-MrNVLP-Dox enhanced the delivery of Dox to HT29 cancer cells expressing high level of folate receptor (FR) as compared to CCD841CoN normal cells and HepG2 cancer cells, which express low levels of FR. As a result, FA-MrNVLP-Dox increased the cytotoxicity of Dox on HT29 cells, and decreased the drug’s cytotoxicity on CCD841CoN and HepG2 cells. This study demonstrated the potential of FA-MrNVLP-Dox as a thermally-responsive nanovehicle for targeted delivery of Dox to cancer cells rich in FR.

## Introduction

Hyperthermia therapy is a form of cancer treatment in which tumour tissues or targeted body parts of cancer patients are exposed to higher temperatures ranging between 39 and 45 °C^1^. Hyperthermia has the property of chemosensitizers, and the treatment is often incorporated into chemotherapy to enhance the sensitivity of cancer cells towards a chemotherapeutic agent^2^.

Novel drug delivery systems which release their payload in response to either internal stimuli (pH, redox, and enzyme concentration) or external stimuli (temperature, light, magnetic field, and ultrasound) have received much attention lately^3^. Thermally-responsive drug delivery systems are stable at the physiological temperature (37 °C) and release their payload in response to elevated temperature, resulting in controlled drug release, enhanced anti-tumour efficacy, and reduced side effects^4^. A variety of nanocarriers such as liposomes, hydrogels, micelles, and dendrimers have been applied in the development of thermally-responsive drug delivery systems^4^. ThermoDox^®^, a thermally-responsive liposome encapsulating doxorubicin (Dox), is currently in phase III clinical trial for the treatment of liver cancer^5^. However, up until now, no information is available on the development of a thermally-responsive drug delivery system based on a virus-like particle (VLP). VLP is a protein shell of a virus without its viral genome. It has many essential qualities as a potential nanoparticle for drug delivery, including (i) biocompatible and biodegradable^6^; (ii) homogenous in size and morphology^6^; (iii) highly ordered structures^7–9^; and (iv) can be functionalised genetically^10,11^ and chemically^12–15^.

*Macrobrachium rosenbergii* nodavirus (MrNV) is a non-enveloped icosahedral virus containing 180 copies of the viral capsid protein^7,16^. Each capsid protein is a single polypeptide comprising 371 amino acids^17^. The recombinant capsid protein expressed in *Escherichia coli* self-assembles into a VLP which encapsidates host RNA molecules^18,19^. This VLP, namely MrNVLP, has been applied in gene delivery^20–22^, development of multi-component vaccines^23,24^, and screening of the viral peptide inhibitors^25^. In addition, Hanapi *et al*.^26^ employed the MrNVLP to study the viral trafficking in insect cells.

Dox is commonly used as a chemotherapy drug to treat various cancers, including breast, lung, ovarian and colorectal cancers^27^. It intercalates DNA, inhibits topoisomerase II, causes cross-linking in DNA strands^28^, and generates reactive oxygen species in cells^29^. The damage of DNA and accumulation of reactive oxygen species lead to arrest of cell cycle and death of tumour cells. However, Dox is highly toxic to normal cells particularly cardiovascular cells^30^. Thus, a specific drug delivery system which can target Dox specifically to cancer cells with reduced side effects on normal cells is urgently needed.

**Figure 1:**
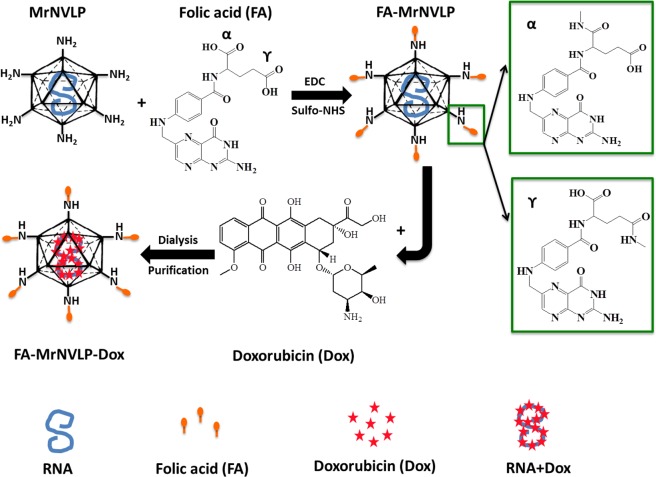
Schematic representation of drug delivery system based on the virus-like particle of *Macrobrachium rosenbergii* nodavirus (MrNVLP). Carboxylic acid groups of folic acid (FA) molecules were conjugated with the primary amines of lysine residues located on the surface of MrNVLP using 1-ethyl-3-(3-dimethylaminopropyl)carbodiimide hydrochloride (EDC) and N-hydroxysulfo-succinimide (sulfo-NHS). The cross-linking generally involves both the alpha (α) and gamma (γ) carboxylic groups of a FA, with the γ-carboxylic group being more accessible for cross-linking due to steric hindrance at the α-carboxylic group^53^. FA molecules conjugated at either α-carboxylic or γ-carboxylic group have the same binding efficiency towards folate receptor (FR) on tumour cells^53^. Doxorubicin (Dox) molecules were infused into the cavity of FA-conjugated MrNVLP (FA-MrNVLP) via interactions with the RNA molecules encapsidated inside the nanoparticle. Excess Dox molecules were removed by dialysis. The FA-conjugated-and-Dox-loaded MrNVLP (FA-MrNVLP-Dox) was purified with sucrose density gradient ultracentrifugation.

Many types of cancer cells including ovary, uterus, kidney, colon, brain and lung cancers over express folic acid receptor (FR) which binds specifically to folic acid (FA) with a high affinity^31,32^. Therefore, FA has been conjugated to many drug delivery nanocarriers to specifically target cancer cells^12–14,33,34^. In this study, we established a Dox delivery system using a novel thermally-responsive nanocarrier based on MrNVLP. The FA-conjugated MrNVLP loaded with Dox, namely FA-MrNVLP-Dox (Fig. 1), recognised colorectal cancer HT-29 cells rich in FR, and released the encapsulated Dox at a hyperthermia temperature (43 °C). The multifunctional nanoparticle established in this study demonstrated specific targeted delivery and thermally-controlled release of Dox.

## Results

### Conjugation of folic acid (FA) to MrNVLP

**Figure 2:**
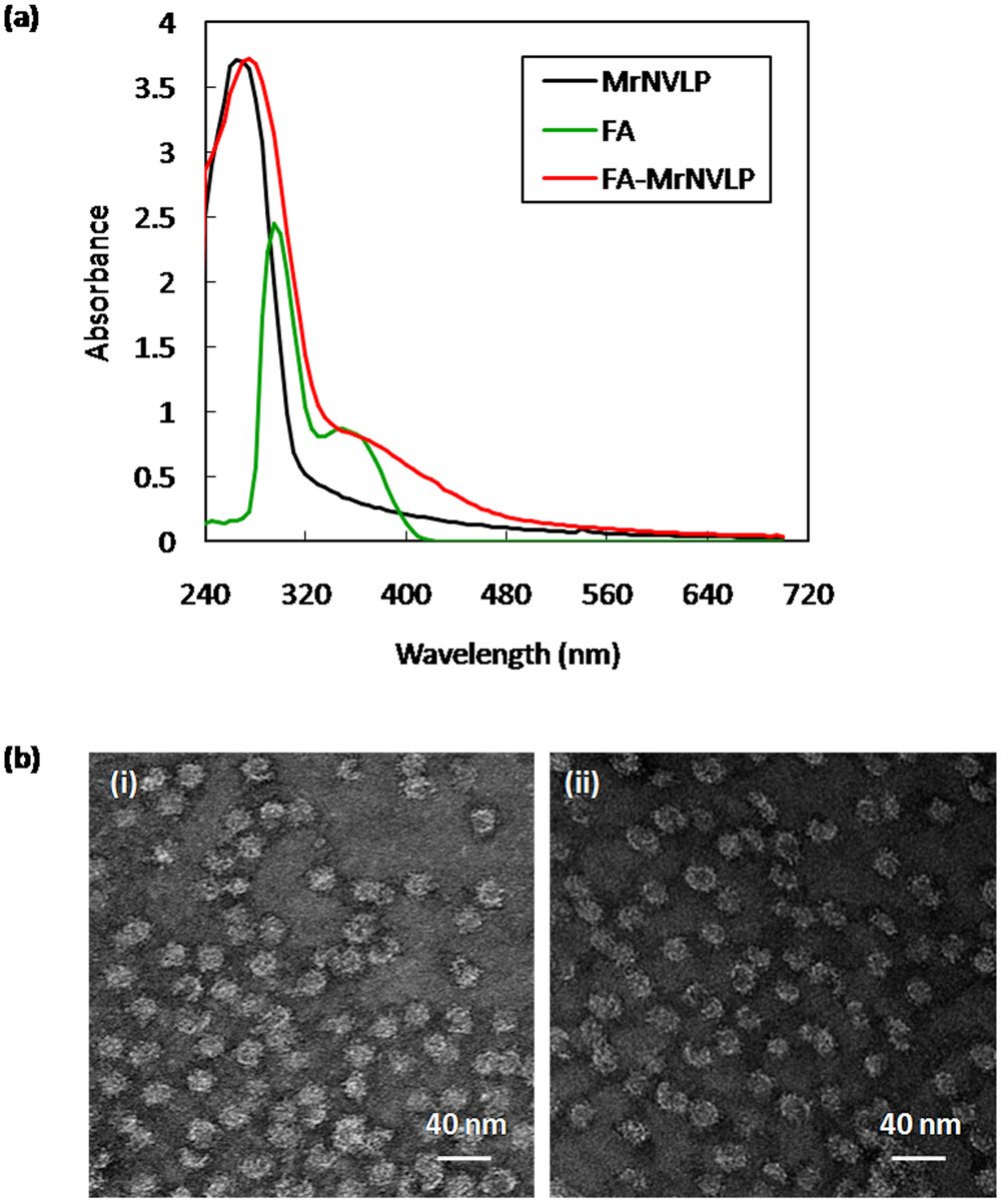
Conjugation of folic acid to MrNVLP. (**a)** UV-visible spectra of the virus-like particle of *Macrobrachium rosenbergii* nodavirus (MrNVLP), folic acid (FA), and FA-conjugated MrNVLP (FA-MrNVLP). **(b)** Transmission electron micrographs of (i) MrNVLP, and (ii) FA-MrNVLP, stained negatively with uranyl acetate.

The carboxylate groups of FAs were covalently conjugated with primary amine groups of lysine residues on the MrNVLP using N-hydroxysulfosuccinimide (sulfo-NHS) and 1-ethyl-3-(3-dimethylaminopropyl)carbodiimide hydrochloride (EDC). The FA-conjugated MrNVLP (FA-MrNVLP) was purified, and its absorbance from wavelength 240 to 700 nm was measured. The result showed that FA-MrNVLP had a higher absorbance at 360 nm compared with MrNVLP (Fig. 2a), indicating FA was successfully conjugated to the MrNVLP. Liquid chromatography-mass spectrometry (LC-MS) detected the mass of MrNV capsid protein at 45333.03 Da (Supplementary Fig. S1a). After FA conjugation, its mass increased to 45738.85 Da and 46015.74 Da, which coressponded well with one and two FAs conjugated to each MrNV capsid protein (Supplementary Fig. S1b). The conjugation efficiency (CE) was 2.0 ± 0.1%, amounting to 377 ± 15 of FAs conjugated to a MrNVLP. Since MrNVLP has an icosahedral structure with a triangulation number *T* = *3*, comprising 180 subunits of the MrNV capsid protein^7,16^, thus approximately one to two FAs were conjugated to each subunit of the MrNV capsid protein. TEM analysis showed that conjugation of FA to MrNVLP did not have an adverse effect on the structure of the VLP (Fig. 2b).

### Loading of doxorubicin (Dox) into MrNVLP

**Figure 3:**
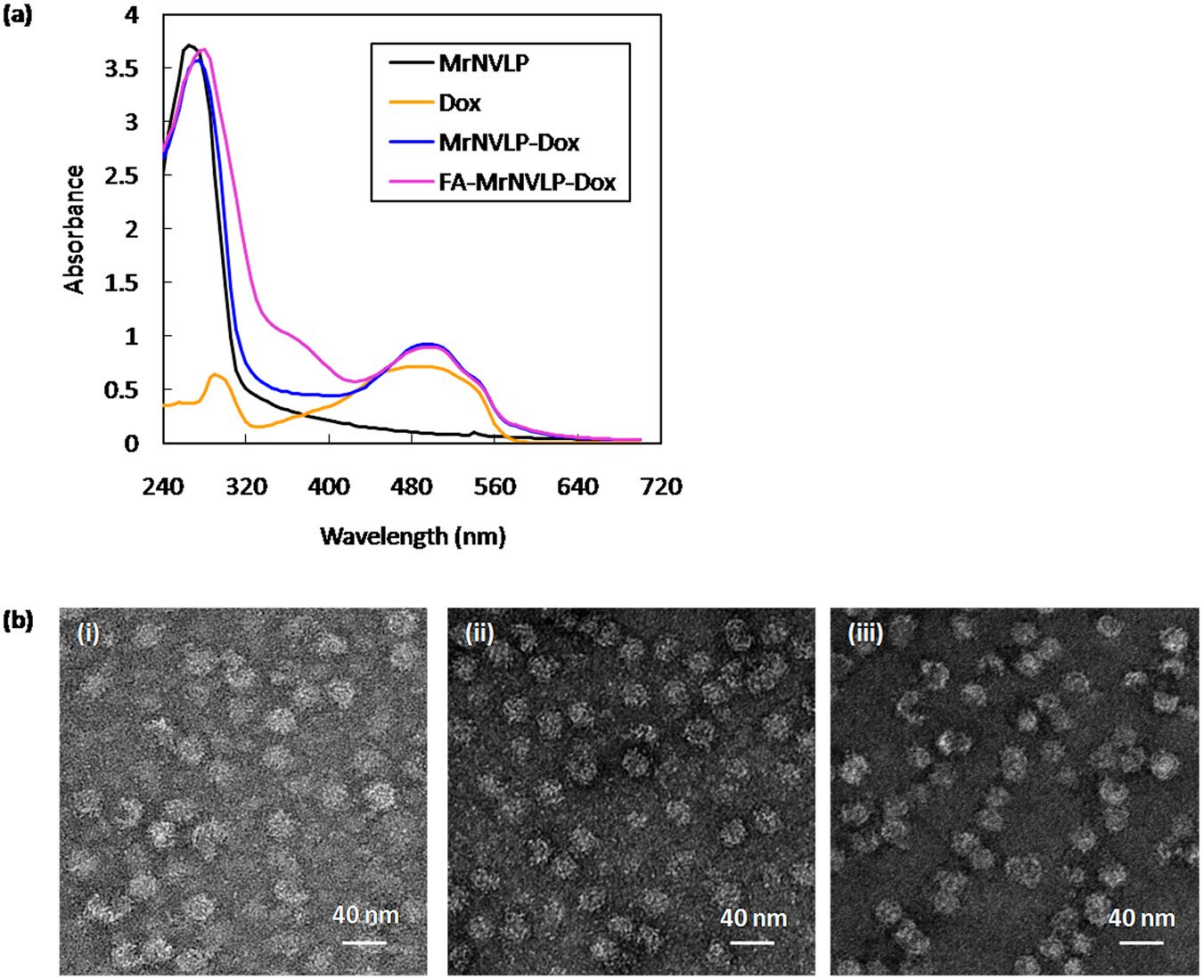
Loading of doxorubicin into MrNVLP. (**a)** UV-visible spectra of the virus-like particle of *Macrobrachium rosenbergii* nodavirus (MrNVLP), free doxorubicin (Dox), Dox-loaded MrNVLP (MrNv-Dox), and Dox-loaded-and-folic acid (FA)-conjugated MrNVLP (FA-MrNVLP-Dox). **(b)** Transmission electron micrographs of (i) MrNVLP, (ii) MrNVLP-Dox, and (iii) FA-MrNVLP-Dox stained negatively with uranyl acetate.

Dox was loaded into MrNVLP using the infusion method as described by Yildiz *et al*.^35^. Figure 3a shows the UV-visible spectra of the MrNVLP loaded with Dox (MrNVLP-Dox) and the FA-conjugated-and-Dox-loaded MrNVLP (FA-MrNVLP-Dox). The MrNVLP-Dox and the FA-MrNVLP-Dox had a higher absorbance at 495 nm as compared with the MrNVLP, indicating the VLPs were loaded with Dox. The entrapment efficiency (EE) of MrNVLP-Dox and FA-MrNVLP-Dox was 5.06 ± 0.29% and 5.47 ± 0.13%, respectively. The loading efficiency (LE) of MrNVLP-Dox and FA-MrNVLP-Dox was 2.1 ± 0.1% and 2.3 ± 0.2%, respectively. The LE values are equivalent to 316 ± 15 and 354 ± 24 of Dox being loaded in a MrNVLP and a FA-MrNVLP, respectively. TEM analysis of the MrNVLP-Dox and FA-MrNVLP-Dox revealed that the VLPs remained intact after being loaded with Dox (Fig. 3b).

### Dynamic light scattering (DLS) and zeta potential of MrNVLP

Dynamic light scattering (DLS) analysis at 25 °C showed that the hydrodynamic radius (R_h_) of the MrNVLP and FA-MrNVLP was 15.54 ± 0.29 and 16.37 ± 0.14 nm, respectively. After the VLPs were loaded with Dox, their R_h_ increased significantly (P < 0.05) to 16.67 ± 0.35 and 17.42 ± 0.35 nm for the MrNVLP-Dox and the FA-MrNVLP-Dox, respectively. The zeta potential value for MrNVLP and MrNVLP-Dox was −8.22 ± 0.19 mV and −8.83 ± 0.52 mV, respectively. Conjugation of FA decreased the amount of protonated amino group on the surface of the nanoparticles, and thus the zeta potential value of FA-MrNVLP and FA-MrNVLP-Dox reduced significantly (P < 0.05) to −11.77 ± 0.80 mV and −11.47 ± 1.31 mV, respectively.

### *In vitro* release of doxorubicin (Dox)

**Figure 4:**
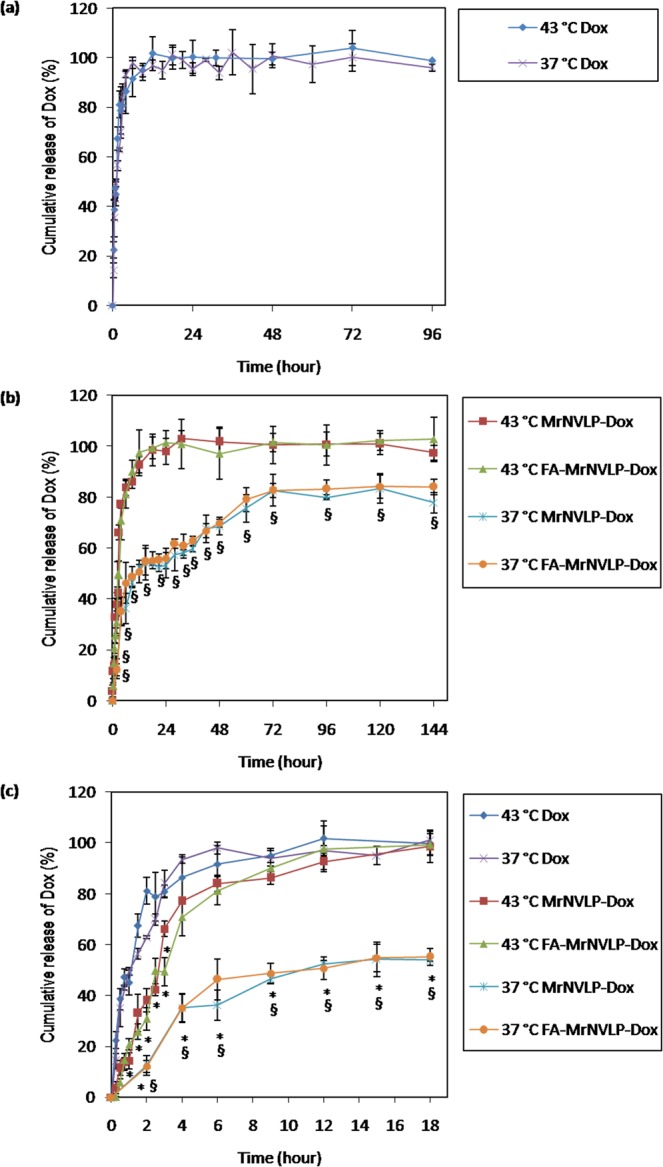
*In vitro* release of doxorubicin loaded inside MrNVLP at different temperatures. Doxorubicin release profile of (**a)** free doxorubicin (Dox), **(b)** virus-like particle of *Macrobrachium rosenbergii* nodavirus loaded with Dox (MrNVLP-Dox), and MrNVLP conjugated with folic acid (FA) and loaded with Dox (FA-MrNVLP-Dox), at the physiological (37 °C) and a hyperthermia temperature (43 °C). **(c)** Drug release profiles of Dox, MrNVLP-Dox, and FA-MrNVLP-Dox for the first 18 h at 37 °C and 43 °C. The rate of drug release of free Dox was not affected at these different temperatures. However, MrNVLP-Dox and FA-MrNVLP-Dox released Dox at a higher rate at 43 °C compared with that at 37 °C. Data are presented as mean ± standard deviation of triplicate measurements. * indicates P < 0.05 when comparing the drug release data of the nanoparticles with the corresponding data points of the free Dox solution. ^§^ indicates P < 0.05 when comparing the drug release data of the nanoparticles at 37 °C with 43 °C.

To study the thermal-responsivity of FA-conjugated-and-Dox-loaded nanoparticles, an *in vitro* drug release experiment was performed at the physiological (37 °C) and hyperthermia (43 °C) temperatures. Figure 4a shows that the rate and the maximum cumulative release of free Dox were not significantly different between 37 °C and 43 °C, indicating the release profile of free Dox was the same at these temperatures; approximately 50% of the free Dox was released into the receptor chamber after 1 h, and almost 100% was released within 18 h. Unlike the free Dox, MrNVLP-Dox and FA-MrNVLP-Dox showed different drug release profiles when the nanoparticles were incubated at 37 °C and 43 °C. These nanoparticles released Dox at a higher rate (P < 0.05) and achieved a higher maximum cumulative release (P < 0.005) when they were incubated at the hyperthermia temperature compared with the physiological temperature (Fig. 4b). At 37 °C, approximately 50% of Dox was released from the nanoparticles after 12 h, and the system only achieved a maximum cumulative release of approximately 80% after 72 h. By contrast, at 43 °C, approximately 50% of Dox was released from the nanoparticles after 3 h, and almost 100% of the Dox was released within 24 h (Fig. 4b). Apart from that, the nanoparticles showed a significantly slower release of Dox as compared with that of the free Dox solution (P < 0.05), demonstrating a more sustained release of drug (Fig. 4c). However, the nanoparticles incubated at 43 °C only showed significantly slower release of Dox (P < 0.05) for the first 3 h of release period, after that the rate of drug release from the nanoparticles was insignificantly different as compared with the free Dox solution (Fig. 4c). These results indicated that the loaded Dox was not released from the nanoparticles during storage before the *in vitro* drug release experiment. In addition, the drug release was performed at pH 5.4 to simulate the microenvironment of tumour tissues and endosomes. The drug release profile at pH 5.4 was not significantly different as compared with that at pH 7.4 (Supplementary Fig. S2), indicating that the thermally responsive property of the nanoparticles was the same at the tested pH.

### Cellular uptake of nanoparticles

**Figure 5:**
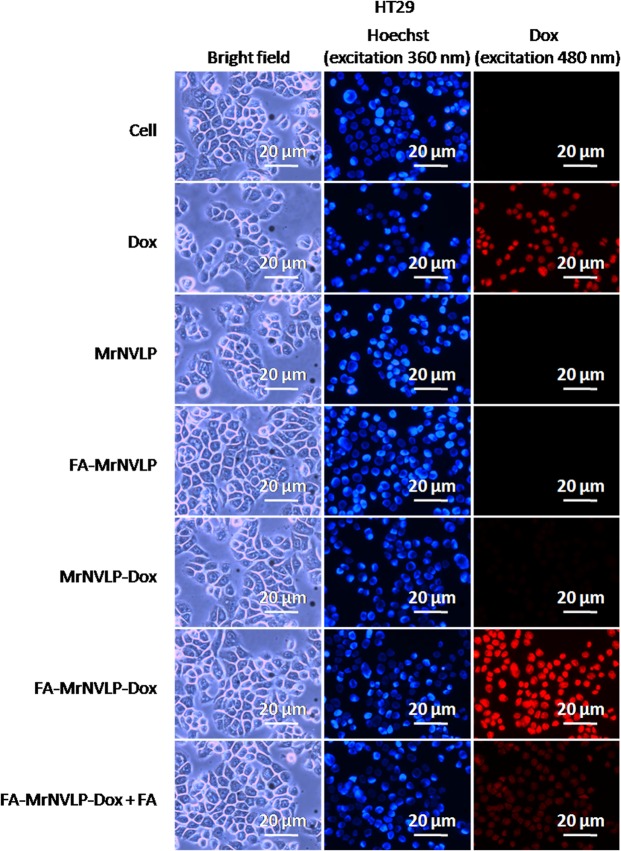
*In vitro* delivery of doxorubicin by MrNVLP into HT29 cells. HT29 cells were incubated with free doxorubicin (Dox), virus-like particle of *Macrobrachium rosenbergii* nodavirus loaded with Dox (MrNVLP-Dox), folic acid (FA)-conjugated-and-Dox-loaded MrNVLP (FA-MrNVLP-Dox), and FA-MrNVLP-Dox added with free FA (FA-MrNVLP-Dox + FA), at equivalent Dox concentration. Untreated cells, cells incubated with MrNVLP, and cells incubated with FA-MrNVLP served as controls.

**Figure 6:**
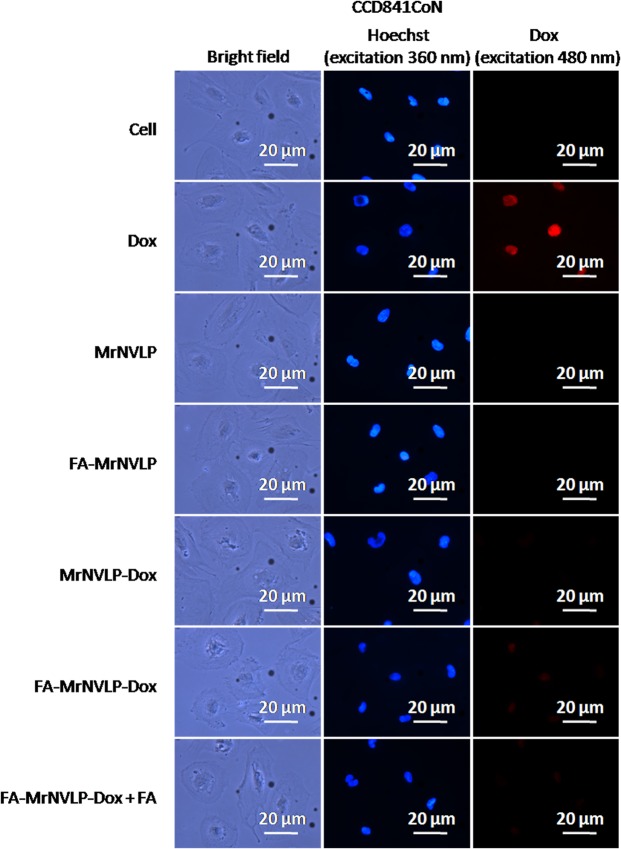
*In vitro* delivery of doxorubicin by MrNVLP into CCD841CoN cells. CCD841CoN cells were incubated with free doxorubicin (Dox), virus-like particle of *Macrobrachium rosenbergii* nodavirus loaded with Dox (MrNVLP-Dox), folic acid (FA)-conjugated-and-Dox-loaded MrNVLP (FA-MrNVLP-Dox), and FA-MrNVLP-Dox added with free FA (FA-MrNVLP-Dox + FA), at equivalent Dox concentration. Untreated cells, cells incubated with MrNVLP, and cells incubated with FA-MrNVLP served as controls.

**Figure 7:**
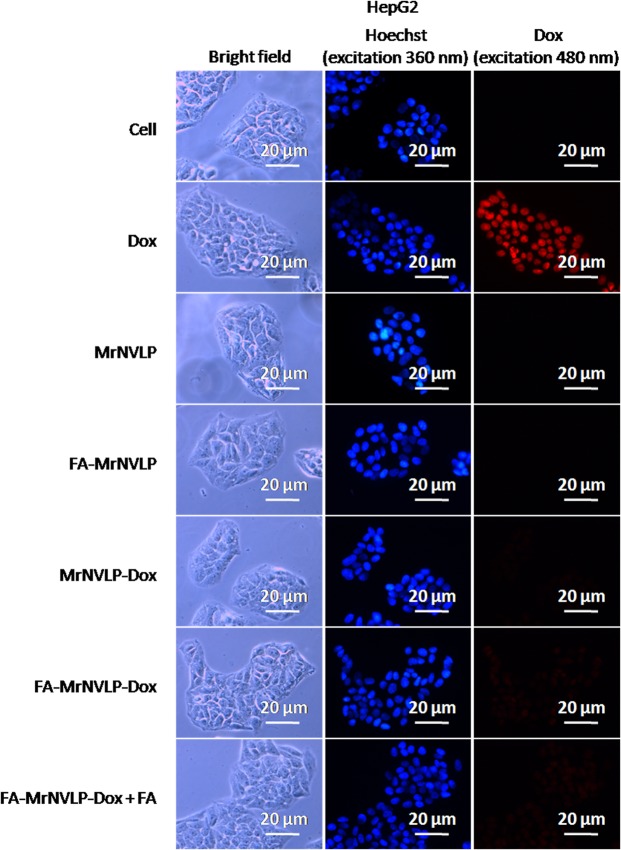
*In vitro* delivery of doxorubicin by MrNVLP into HepG2 cells. HepG2 cells were incubated with free doxorubicin (Dox), virus-like particle of *Macrobrachium rosenbergii* nodavirus loaded with Dox (MrNVLP-Dox), folic acid (FA)-conjugated-and-Dox-loaded MrNVLP (FA-MrNVLP-Dox), and FA-MrNVLP-Dox added with free FA (FA-MrNVLP-Dox + FA), at equivalent Dox concentration. Untreated cells, cells incubated with MrNVLP, and cells incubated with FA-MrNVLP served as controls.

Cellular uptake of the free Dox, MrNVLP-Dox, and FA-MrNVLP-Dox was analysed using HT29 colorectal cancer cell line over expressing FR^36^, CCD841CoN colorectal normal cell line and HepG2 liver cancer cell line expressing low levels of FR^37,38^. HT29 (Fig. 5), CCD841CoN (Fig. 6), and HepG2 (Fig. 7) cells treated with free Dox exhibited an intense red fluorescence in nuclei, demonstrating a non-specific cellular uptake of Dox. HT29 cells incubated with the MrNVLP-Dox showed a much lower intensity of red fluorescence compared to those incubated with free Dox, but HT29 cells incubated with the FA-MrNVLP-Dox showed a higher intensity of red fluorescence in nuclei (Fig. 5), demonstrating the conjugation of FA increased the uptake of Dox loaded inside the nanoparticles by HT29 cells over expressing FR. CCD841CoN and HepG2 cells incubated with the MrNVLP-Dox and FA-MrNVLP-Dox, showed a much lower fluorescence intensity than the same cells incubated with free Dox (Figs 6 and 7), demonstrating that the uptake of Dox by cells which do not over express FR was lower when the drug was packaged inside the nanoparticles. To further assess the role of FA in cellular targeting, the red fluorescence intensity of cells incubated with the FA-MrNVLP-Dox in the absence and presence of free FA were compared. The presence of free FA in culture media did not affect the red fluorescence intensities of the CCD841CoN (Fig. 6) and the HepG2 (Fig. 7) cells which were incubated with FA-MrNVLP-Dox. However, the red fluorescence intensity of HT29 cells incubated with the FA-MrNVLP-Dox decreased when FA was added in the culture media (Fig. 5), indicating a competition between free FA and FA-MrNVLP-Dox for FR on the surface of HT29 cells. The result indicates that the uptake of FA-MrNVLP-Dox by HT29 cells was mediated by FR. Untreated cells, cells incubated with MrNVLP, and cells incubated with FA-MrNVLP, which served as negative controls, did not show red fluorescence when they were excited at wavelength 480 nm (Figs 5, 6 and 7).

### Cytotoxicity of various doxorubicin (Dox) formulations

**Figure 8:**
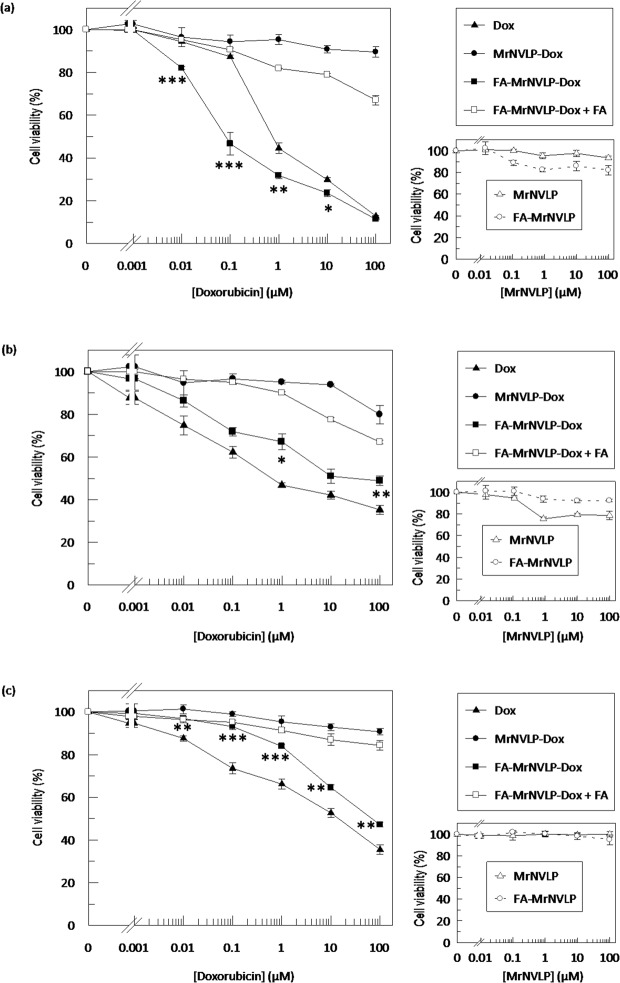
Cytotoxicity of various doxorubicin formulations. Viability of **(a)** HT29 **(b)** CCD841CoN, and **(c)** HepG2 cells after being treated with free doxorubicin (Dox) and various nanoparticles that carried equal Dox concentration. Dox-loaded in the virus-like particle of *Macrobrachium rosenbergii* nodavirus (MrNVLP-Dox) was less cytotoxic to all the tested cell lines as compared with free Dox. MrNVLP conjugated with folic acid (FA) and loaded with Dox (FA-MrNVLP-Dox) was more cytotoxic to HT29 cells compared with free Dox. Conversely, FA-MrNVLP-Dox exhibited a lower cytotoxicity to CCD841CoN and HepG2 cells as compared with free Dox. Small graphs on the right show that MrNVLP and FA-conjugated MrNVLP (FA-MrNVLP) were not cytotoxic to the tested cells. Data are expressed as mean ± standard deviation of triplicate measurements. *** indicates P < 0.001, ** indicates P < 0.005, and *indicates P < 0.05 when comparing the cytotoxicity of FA-MrNVLP-Dox with free Dox.

The cytotoxicity of various Dox formulations towards HT29, CCD841CoN, and HepG2 cells was assessed using the MTT assay. IC_50_ value could not be determined when all the three cell lines were treated with MrNVLP-Dox, indicating the cytotoxicity of Dox was reduced when it was loaded inside the MrNVLP. The IC_50_ values of free Dox and FA-MrNVLP-Dox for HT29 cells were 0.89 ± 0.07 µM and 0.18 ± 0.14 µM respectively (Fig. 8a), showing that the FA-MrNVLP-Dox was significantly more potent than free Dox in inhibiting HT29 cells (P < 0.001). The presence of free FA in culture media substantially reduced the cytotoxicity of FA-MrNVLP-Dox towards HT29 cells (Fig. 8a). This observation correlates well with the result of cellular uptake of HT29 cells (Fig. 5) which further supports the role of FA in cellular targeting. The IC_50_ values of free Dox and FA-MrNVLP-Dox for CCD841CoN cells were 0.82 ± 0.08 µM and 44.55 ± 26.40 µM, respectively (Fig. 8b). The IC_50_ values of free Dox and FA-MrNVLP-Dox for HepG2 cells were 13.19 ± 5.81 µM and 86.10 ± 4.89 µM, respectively (Fig. 8c). This reveals that the FA-MrNVLP-Dox was significantly less toxic to CCD841CoN and HepG2 cells compared with free Dox (P < 0.001). The negative controls, MrNVLP and FA-MrNVLP, did not show cytotoxic effects on the tested cell lines (Fig. 8).

### Apoptosis of HT29 cells

Flow cytometry with FITC annexin V staining was applied to determine apoptosis in HT29 cells. Apoptotic cells bound to FITC annexin V and showed higher intensity of fluorescence signals (Supplementary Fig. S3a). Percentage of apoptotic cells (Supplementary Fig. S3b) was determined by comparing the ratio of cells shifted to higher fluorescence signal to the total amount of cells (Supplementary Fig. S3a). For HT29 cells added with MrNVLP and incubated at hyperthermia temperature (43 °C) for 30 min, the amount of apoptotic cells did not increase as compared to the non-treated control cells. Treatment with Dox alone showed 36.93 ± 0.34% of apoptotic (annexin V-positive) HT29 cells. Hyperthermia incorporated Dox treatment showed 38.21 ± 0.74% of apoptotic cells, which was not significantly different with the treatment of Dox alone. Treatment of FA-MrNVLP-Dox resulted in 47.98 ± 2.71% of apoptotic cells, which was significantly higher compared with the treatment of Dox alone (P < 0.01). This result corresponded well with the MTT assay, indicating FA-MrNVLP-Dox was more cytotoxic to HT29 cells as compared with free Dox. Interestingly, hyperthermia incorporated FA-MrNVLP-Dox treatment significantly increased the percentage of apoptotic cells to 62.01 ± 1.72% as compared with the treatment of FA-MrNVLP-Dox alone (P < 0.01), demonstrating the thermally responsive FA-MrNVLP-Dox induced greater apoptosis when hyperthermia was incorporated in the treatment.

Apoptosis in mammalian cells is initiated with activation of caspases. In this study, caspase-9 activity was assayed to verify apoptosis in HT29 cells (Supplementary Fig. S3c). HT29 cells incubated with MrNVLP, and the cells treated with hyperthermia alone did not show an increase in caspase-9 activity compared with non-treated cells. Cells treated with free Dox significantly increased the caspase-9 activity to 1.04 ± 0.05 fold compared with non-treated cells (P < 0.001). Treatment with free Dox plus hyperthermia increased the caspase-9 activity to 1.07 ± 0.07 fold, which was not significantly different as compared with the treatment of free Dox alone without incorporation of hyperthermia. Cells incubated with FA-MrNVLP-Dox showed 1.26 ± 0.03 fold increase in caspase-9 activity, demonstrating a significantly higher apoptosis compared with cells treated with free Dox (P < 0.01). HT29 cells treated with FA-MrNVLP-Dox in addition of hyperthermia showed the highest increment in caspase-9 activity, which was 1.48 ± 0.06 fold. These results corresponded well with the data of FITC annexin V staining.

## Discussion

Chemotherapy is commonly used to treat cancer patients. However, this treatment has many side effects due to non-specific distribution of therapeutic drugs to normal cells. These side effects can be minimised by using cell specific nanocarriers such as liposomes, polymers, and VLPs. Currently, multifunctional VLPs displaying specific ligand and simultaneously response to stimuli have gained more attention in specific targeted drug delivery. For instance, the VLP of hepatitis B virus displaying FA that responses to pH changes has been applied to deliver Dox into colorectal cancer cells^12^. However, no information is available on thermally-responsive VLP in drug delivery system. Hence, the aim of this study was to develop a thermally-responsive drug delivery system using VLP. In the present study, FA was conjugated to the primary amines exposed on the surface of MrNVLP using EDC and sulfo-NHS (Fig. 1). Dox was then infused into the cavity of FA-MrNVLP via interactions with RNA molecules (Fig. 1). Cowpea mosaic virus^35^, cucumber mosaic virus^39^, and red clover necrotic mosaic virus^40^ were used to package cationic molecules by electrostatic interactions with the anionic nucleic acids encapsidated inside the cavity of the viral particles. In addition, Dox interacts with the major and minor grooves of RNA molecules via hydrophilic and hydrophobic forces^41^. MrNVLP encapsidates host RNA molecules when the viral capsid protein was expressed in *Escherichia coli*^18,19^. Cryo-electron microscopy and image reconstruction of MrNVLP at 3.3 Å resolution^16^ revealed that the VLP contains pores which allow small molecules such as Dox to infuse into the particle. Besides, the packaged RNA molecules appeared as a dodecahedral cage structure^7,16^. Therefore, we adapted the method described by Yildiz *et al*.^35^ to load Dox into MrNVLP. Dox was incubated with MrNVLP, allowing the drug to infuse into the cavity of MrNVLP and retain inside the VLP by interactions with the RNA molecules. Excess Dox molecules were removed by dialysis, and the MrNVLP loaded with Dox was then purified using sucrose density gradient ultracentrifugation. Approximately 350 Dox molecules were loaded inside each MrNVLP, which was confirmed spectrophotometrically and by DLS analysis. This simple and time-saving infusion method has several advantages over the disassembly-and-reassembly packaging method and the covalent conjugation method. The infusion method preserves the stability of MrNVLP and the bioactivity of Dox as both the vehicle and cargo are not modified. In contrast, the disassembly-and-reassembly packaging method involves dissociation and re-association of VLPs, hence may affect the stability of VLPs and some protein subunits may not re-assemble properly in their native structures^42^. Whereas in covalent conjugation method, the chemical structure of drug is altered in order to covalently link to VLPs and this may affect bioactivity of the conjugated drug^14^. In addition, conjugation of drugs on VLPs may also affect the solubility of the conjugated products^14^.

In this study, the Dox loading efficiency of MrNVLP was approximately 2%. The loading efficiency of Dox in other VLPs such as those of hepatitis B core antigen (HBcAg)^12^, cucumber mosaic virus^39^, and Rous sarcoma virus^11^ was approximately 13%, 14% and 0.7%, respectively. Although the loading efficiency was rather low, *in vitro* and *in vivo* drug delivery using these VLPs yieled promising results, indicating the potential of VLPs in drug delivery.

The first thermally-responsive nanocarrier was introduced by Yatvin and colleagues in 1978^43^. Since then, several smart thermally-responsive nanocarriers have been proposed^44^. Thermally-responsive nanocarriers protect the encapsulated drug at physiological temperature and hyperthermia triggers the release of the encapsulated drug from the nanocarriers, enabling controlled drug release^44^. In the present study, the rate of Dox released from MrNVLP was considerably higher at 43 °C compared with that at 37 °C, possibly due to a higher degree of loosening of MrNVLP at 43 °C. As determined by DLS analysis, the R_h_ of MrNVLP was 15.55 ± 0.48 nm at 37 °C, whereas the R_h_ increased significantly to 19.02 ± 1.22 nm at 43 °C (P < 0.01). Needham *et al*.^45^ also reported a similar observation by using the ThermoDox^®^; a temperature-sensitive liposome which is stable at 37 °C and releases encapsulated Dox at 40 °C. Apart from that, *in vitro* drug release profile for MrNVLP-Dox and FA-MrNVLP-Dox nanoparticles at 37 °C showed a biphasic release pattern, with a faster release in an initial period of 6 h, followed by a slower, sustained release up to 6 days. The initial release of drug from these nanoparticles may be caused by the release of Dox molecules that interact with the phosphate groups of nucleic acids via weak electrostatic force (ΔG = −1.0 kcal mol^−1^)^46^. The sustained release thereafter could be due to Dox molecules that interact with RNA molecules via a stronger hydrophilic and hydrophobic forces (ΔG = −4.4 kcal mol^−1^)^41^. The two kinetic process of drug release at 37 °C was not observed at 43 °C. At this hyperthermia temperature, it is believed that protein-protein and protein-RNA interactions of the nanoparticles are weakened, causing water molecules flux from exterior of the nanoparticles into their cavity. This process would increase the internal pressure of the nanoparticles, followed by swelling and bursting, giving rise to one phase and higher rate of drug release. Zandi and Reguera^47^, as well as Pérez-Berná^48^ demonstrated that icosahedral viral capsids swelled and their internal pressure increased at high temperatures, which weakened protein-protein interactions of capsomers. As a result, the capsids cracked at hexameric position, and led to capsid bursting. In the apoptotic experiments, HT29 cells treated with FA-MrNVLP-Dox were incubated at 43 °C for 30 min to determine the effect of hyperthermia therapy of the thermally responsive nanoparticles. A longer period of hyperthermia therapy could be applied in an *in vivo* system particularly at the specific tumour site. In addition, as a prototype of thermally-responsive VLP in cancer drug delivery, the potential of MrNVLP in hyperthermia therapy could be further exploited by a variety of established heating methods such as ultrasound transducer, radiofrequency, and near-infrared laser.

Conjugation of FA to nanoparticles loaded with chemotherapeutic drugs such as Dox^12–14^, docetaxel^49^, and 5-fluorouracil^50^ enhanced the delivery of these drugs to cancer cells and reduced their cytotoxicity on normal cells. FR is over expressed in many types of cancer cells compared to normal cells^36^. Besides, cancer cells recognise FA or FA-conjugates through FR, however normal cells uptake 5-methyltetrahydrofolate (a reduced form of FA) through the reduced folate carrier (RFC)^51^. As such, FA-conjugated nanoparticles can internalise cancer cells at a higher rate than normal cells. This is in line with our finding in which FA-MrNVLP-Dox was taken up at a higher level by the colorectal cancer HT29 cells expressing high level of FR compared with the colorectal normal CCD841CoN cells expressing low level of FR. Moreover, HepG2, a hepatocellular carcinoma cell line expressing low level of FR, also demonstrated a lower uptake of FA-MrNVLP-Dox compared with the HT29 cells. This result confirmed that the delivery of FA-MrNVLP-Dox to HT29 cells was mediated by FR.

In summary, we established a novel method for direct loading of Dox into MrNVLP. Conjugation of FA on the VLP increased the cellular uptake and accumulation of Dox in HT29 colorectal cancer cells which are rich in FR. Furthermore, the FA-MrNVLP-Dox also enhanced the toxicity of Dox and increased the apoptotic activity on HT29 cells. Apart from FA, other tumour-targeting ligands could also be displayed on MrNVLP using appropriate cross-linkers. In addition, other positively charged molecules or nucleic acid binding molecules could be loaded in the MrNVLP via the infusion method. The sustained-release profile and a nearly complete release of all the loaded Dox at hyperthermia temperature demonstrate the potential of MrNVLP as an appealing candidate for thermally-responsive drug delivery system.

## Materials and Methods

### Conjugation of folic acid (FA) to MrNVLP

Expression and purification of MrNVLP were performed as described by Goh *et al*.^18^. The carboxylate group of FA was activated by incubating FA (4 mM) with EDC (2.4 mM) and sulfo-NHS (2.4 mM) in HEPES buffer (25 mM HEPES, 150 mM NaCl, pH 7.4) for 2 h at 25 °C. The mixture (1 mL) was then incubated with the purified MrNVLP (1 mL, 3 mg/mL in HEPES buffer, pH 7.4) for 2 h at 4 °C. Next, the mixture was dialysed in HEPES buffer (1 L, two times) at 4 °C to remove excess FA, EDC, and sulfo-NHS. The dialysed mixture (1 mL) was layered on top of a sucrose density gradient [8–50% (w/v)] and centrifuged at 150,000 × g for 4.5 h at 4 °C. The sucrose gradient was fractionated and the concentration of FA-MrNVLP in each fraction was determined using the Bradford assay^52^. The fractions that contained FA-MrNVLP were pooled, dialysed in HEPES buffer at 4 °C, and concentrated with the Vivaspin Turbo 15 (10 kDa cut off, polyethersulfone membrane; Sartorius, Germany). UV-visible measurement of MrNVLP and FA-MrNVLP was performed using a spectrophotometer (Jenway 7315, Cole-Parmer, U.K.). A_360_ and an extinction coefficient of 5312 cm^−1^ M^−1^ were used to calculate the FA conjugation efficiency (CE) using equation (1). The number of FA per MrNVLP (N_FA_) was calculated using equation (2).

1 $${\rm{CE}} \% ={{\rm{Weight}}}_{{\rm{FA}}}/{{\rm{Weight}}}_{{\rm{MrNVLP}}}\times 100 \% $$

2 $${{\rm{N}}}_{{\rm{FA}}}={\rm{CE}}\times ({{\rm{MW}}}_{{\rm{MrNVLP}}}/{{\rm{MW}}}_{{\rm{FA}}})$$

MW; molecular weight.

### Liquid chromatography-mass spectrometry (LC-MS)

MrNVLP and FA-MrNVLP were separated on a SDS-12% polyacrylamide gel by electrophoresis at 16 mA. The capsid protein bands were excised and homogenized in a micro-centrifuge tube. SDS elution buffer [0.25 M Tris-HCl, 0.05% (w/v) SDS; pH 6.8] was added to the tube and vortexed. The mixture was transferred to a Vivaspin Turbo 15 (300 kDa cut off, polyethersulfone membrane; Sartorius, Germany) and centrifuged at 4000 × g for 20 min at 4 °C. The filtrate was desalted using the Vivaspin Turbo 15 (10 kDa cut off, polyethersulfone membrane; Sartorius, Germany). The samples were then analysed on the Agilent 1290 Infinity liquid chromatography (LC) system (Germany) coupled to an Agilent 6520 Accurate-Mass Q-TOF mass spectrometer with a dual ESI source (U.S.A.). The samples were injected into an Agilent Zorbax 300SB-C18 Narrow-Bore (2.1 × 150 mm, 5 µm) column (U.S.A.) with an injection volume of 1 µL. The mobile phase consisted of 0.1% formic acid in water (A) and 0.1% formic acid in acetonitrile (B). A gradient elution was performed by adding B into A at a flow rate of 0.5 mL/min as follows: initially, 5% B; 5 min, 5% B; 20 min, 5–100% B; and 25 min, 100% B. Nitrogen was used as the sheath gas. The capillary temperature and the voltage were set at 300 °C and 4 kV, respectively. The data were analysed using the Agilent MassHunter Qualitative Analysis B.05.00 software.

### Loading of doxorubicin (Dox) into MrNVLP

MrNVLP (7 mg/mL) and FA-MrNVLP (7 mg/mL) were incubated separately with Dox (0.5 mg/mL) in HEPES buffer for 3 h at 25 °C, and then for 16 h at 4 °C. The mixtures (1 mL each) were dialysed in HEPES buffer (1 L, two times) at 4 °C to remove excess Dox. The Dox-loaded MrNVLP (MrNVLP-Dox) and MrNVLP loaded with Dox and conjugated with FA (FA-MrNVLP-Dox) were purified from the mixtures using sucrose density gradient ultracentrifugation as described in the above section. Control samples comprising MrNVLP and free Dox were also analysed separately using sucrose density gradient ultracentrifugation. The sucrose gradients were then fractionated (300 µL) and the presence of Dox in each fraction was analysed at A_490_ using a microtiter plate reader (ELx800, Bio-Tek Instruments, U.S.A.). The concentration of MrNVLP in each fraction was determined using the Bradford assay^52^. The fractions that contained Dox and MrNVLP were pooled, dialysed in HEPES buffer (1 L, two times) at 4 °C, and concentrated with the Vivaspin Turbo 15 (10 kDa cut off, polyethersulfone membrane; Sartorius, Germany). A_495_ and an extinction coefficient of 8030 cm^−1^ M^−1^ were used to calculate the entrapment efficiency (EE), loading efficiency (LE), and the number of Dox per MrNVLP (N_Dox_) using equations (3), (4), and (5), respectively.

3 $${\rm{EE}} \% ={{\rm{Weight}}}_{{\rm{doxorubicin}}{\rm{loaded}}}/{{\rm{Weight}}}_{{\rm{total}}{\rm{doxorubicin}}{\rm{used}}}\times 100 \% $$

4 $${\rm{LE}} \% ={{\rm{Weight}}}_{{\rm{doxorubicin}}{\rm{loaded}}}/{{\rm{Weight}}}_{{\rm{MrNVLP}}}\times 100 \% $$

5 $${{\rm{N}}}_{{\rm{Dox}}}={\rm{LE}}\times ({{\rm{MW}}}_{{\rm{MrNVLP}}}/{{\rm{MW}}}_{{\rm{doxorubicin}}})$$

MW; molecular weight.

### Dynamic Light Scattering (DLS)

The sizes of nanoparticles formed by MrNVLP, FA-MrNVLP, MrNVLP-Dox, and FA-MrNVLP-Dox were determined with a dynamic light scattering (DLS) machine (DynaPro-801^TM^, Protein Solution, U.K.). The purified nanoparticles (250 µg/mL in HEPES buffer, pH 7.4) were injected into a far UV quartz cuvette (Hellma, Germany) and placed in the DLS machine. The hydrodynamic radii (R_h_) of the nanoparticles were measured as described by Goh *et al*.^18^.

### Zeta potential measurement

The purified nanoparticles (250 µg/mL in HEPES buffer, pH 7.4) were loaded into disposable folded capillary cells (DTS1070, Malvern, U.K.). Then, the zeta potential of the samples was determined by using a Zetasizer Nano ZS (Malvern, U.K.) instrument at 4 °C.

### Transmission electron microscopy

MrNVLP, FA-MrNVLP, MrNVLP-Dox, and FA-MrNVLP-Dox (10–40 µg/mL each; 15 µL) were adsorbed onto 300-mesh carbon/formvar-coated copper grids. The particles were stained negatively with freshly prepared and filtered uranyl acetate solution [2% (w/v) in distilled water; 15 µL] for 5 min. The grids were viewed under a TEM (Hitachi H-7700, Japan).

### Cell culture

The human colorectal cancer cell line (HT29) was cultured in FA-deficient Dulbecco’s Modified Eagle’s Medium (DMEM; Sigma Aldrich, U.S.A.) supplemented with 10% (v/v) foetal bovine serum (FBS; Sigma Aldrich, U.S.A.). The human colorectal normal cell line (CCD841CoN) and the human liver cancer cell line (HepG2) were grown in Eagle’s Minimum Essential Medium (EMEM; American Type Culture Collection, ATCC, U.S.A.) containing 10% (v/v) FBS. All cell lines were cultured at 37 °C in a humidified atmosphere of 5% CO_2_ and 95% air.

### *In vitro* release of doxorubicin (Dox)

Drug release experiment was performed using the dialysis method^12,13^, with some modifications. Free Dox (1 mL), MrNVLP-Dox (1 mL), and FA-MrNVLP-Dox (1 mL), with an equivalent concentration (360 µg/mL) of Dox, were placed in individual dialysis tubes (12 kDa cut off; Sigma-Aldrich, U.S.A.) and dialysed in phosphate-buffered saline (PBS; 30 mL; 10 mM Na_2_HPO_4_, 1.8 mM KH_2_PO_4,_ 137 mM NaCl, 2.7 mM KCl, pH 7.4) with gentle agitation at 37 °C and 43 °C. A set of samples were dialysed in PBS at pH 5.4. At each set time point, an aliquot of dialysis buffer (1 mL) was collected for A_495_ measurement to quantify the released Dox. After each collection, PBS (1 mL, pH 7.4) was added to the dialysis buffer in order to replace the removed volume.

### *In vitro* cellular uptake of nanoparticles

HT29, HepG2 and CCD841CoN cell lines were grown on glass coverslips in 6-well plates. The cells were incubated with media (1 mL) containing free Dox, MrNVLP-Dox or FA-MrNVLP-Dox with an equivalent concentration (5 µg/mL) of Dox for 1 h, followed by washing with PBS. The cells were then fixed with paraformaldehyde [3.7% (w/v) in PBS] for 10 min at 25 °C, and washed with PBS. The nuclei were stained with Hoechst 33342 (2 drops/mL in PBS; Life Technologies, U.S.A.) for 10 min at 25 °C. After washing with PBS, the coverslips were mounted on glass slides and viewed under a fluorescence microscope (Olympus X5, Japan). The cells incubated with FA-MrNVLP-Dox in media containing free FA (4 µg/mL) were used to study the role of FA in cellular uptake. Untreated cell lines, MrNVLP, and FA-MrNVLP were used as negative controls.

### Cytotoxicity of doxorubicin (Dox) loaded inside MrNVLP

Cytotoxicity of free Dox, MrNVLP-Dox, and FA-MrNVLP-Dox was evaluated using the MTT cell viability assay. HT29 (2.0 × 10^4^ cell/well), HepG2 (2.0 × 10^4^ cell/well), and CCD841CoN (3.0 × 10^4^ cell/well) cells were seeded in 96-well plates. After 24 h, the culture media were aspirated, and the cells were added with media (100 µL) containing free Dox, MrNVLP-Dox or FA-MrNVLP-Dox at 10-fold serial dilutions (0.001–100 µM), and incubated for 3 h. Then, the solution in each well was replaced with fresh media without Dox (100 µL), and the cells were incubated for 72 h. After that, MTT reagent (5 mg/mL in PBS; 20 µL) was added to the wells and incubated for 3 h. Dimethyl sulfoxide (DMSO; 100 µL) was then added to the wells to dissolve the formazan crystal formed by viable cells. A_570_ was measured using a microtiter plate reader (ELx800, Bio-Tek Instruments, U.S.A.). The cells incubated with FA-MrNVLP-Dox in media containing free FA (4 µg/mL) were used to study the effect of free FA on the cytotoxicity of FA-MrNVLP-Dox. The cells incubated with MrNVLP and FA-MrNVLP were used as negative controls. Untreated cells were used to indicate 100% viability.

### Determination of apoptotic activity of HT29 cells

HT29 (2.0 × 10^5^ cell/well) cells were seeded in 6-well plates. After 24 h, the culture media were aspirated, and the cells were added with media (2 mL) containing free Dox or FA-MrNVLP-Dox at equivalent concentration of Dox (5 µM) and incubated for 3 h. Then, the solution in each well was replaced with fresh media without Dox (2 mL), and the cells were incubated at 43 °C for 30 min, followed by incubation at 37 °C for 71.5 h. For controls, Dox and FA-MrNVLP-Dox treated HT29 cells without incubation at 43 °C, were incubated at 37 °C for 72 h. Apoptotic activity of the cells was then determined by using the FITC Annexin V Apoptosis Detection Kit (BD BioSciences, U.S.A.). The cells were stained by FITC annexin V according to the manufacturer’s protocol and the fluorescence signal was determined by a flow cytometer (NovoCyte, ACEA Biosciences, U.S.A.). The data were analysed using the NovoExpress Software (ACEA Biosciences, U.S.A.). Caspase-9 activity of cells treated as above was quantified using the Caspase-9 Colorimetric Asssay Kit (BioVision, U.S.A.) by following the manufacturer’s manual. The caspase-9 activity was calculated as fold increment compared with the non-treated cells.

### Statistical analysis

Significant differences between tested groups were determined with the one-way ANOVA and Tukey’s HSD test, using the SPSS software. P values of less than 0.05 were considered significant.

## Supplementary information

Supplementary Information

## Data Availability

All data generated or analysed during this study are included in this published article and its Supplementary Information file.
