# Supplementary material for: Thermally-responsive Virus-like Particle for Targeted Delivery of Cancer Drug
Source: Sci Rep. 2019 Mar 8;9:3945. doi: 10.1038/s41598-019-40388-x (PMC6408444; doi:10.1038/s41598-019-40388-x)
Supplement: Supplementary file 1 — Supplementary Information [file 41598_2019_40388_MOESM1_ESM.pdf]

Supplementary Information

Thermally-responsive Virus-like Particle for Targeted Delivery of Cancer Drug

Qiu Xian Thong[1], Roya Biabanikhankahdani[1,2], Kok Lian Ho[3], Noorjahan Banu Alitheen[4,5], and Wen Siang Tan[1,5,*]

[1]Department of Microbiology, Faculty of Biotechnology and Biomolecular Sciences, Universiti Putra Malaysia, 43400 UPM Serdang, Selangor, Malaysia.

[2]Department of Microbiology, College of Science, Agriculture and Modern Technologies, Shiraz Branch, Islamic Azad University, Shiraz, Iran.

[3]Department of Pathology, Faculty of Medicine and Health Sciences, Universiti Putra Malaysia, 43400 UPM Serdang, Selangor, Malaysia.

[4]Department of Cell and Molecular Biology, Faculty of Biotechnology and Biomolecular Sciences, Universiti Putra Malaysia, 43400 UPM Serdang, Selangor, Malaysia.

[5]Institute of Bioscience, Universiti Putra Malaysia, 43400 UPM Serdang, Selangor, Malaysia.

[*]Correspondence and requests for materials should be addressed to W.S.T. (email:wstan@upm.edu.my)

(a)

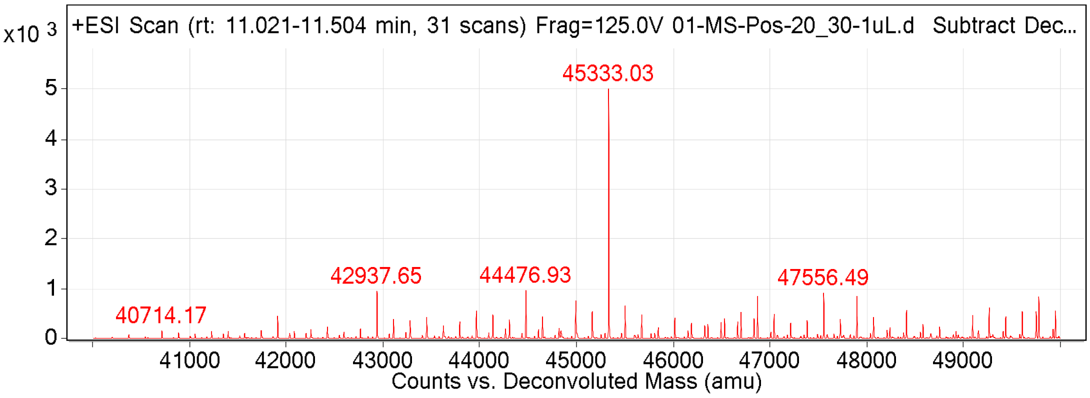

MS Spectrum Peak List

m/z	Abundance
42937.65	953.97
44476.93	970.39
44992.84	755.95
45333.03	5016.46
45503.02	651.40
46872.00	847.21
47556.49	910.95
47898.66	852.51
49266.64	625.91
49779.65	839.91

(b)

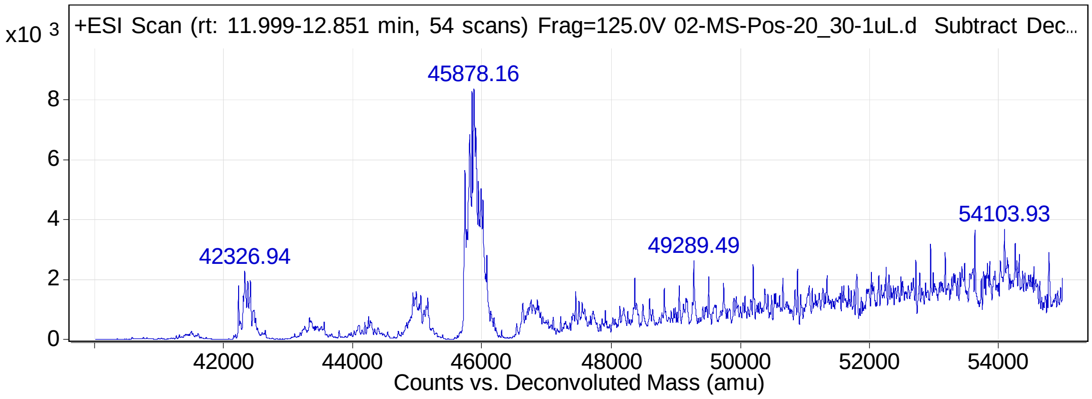

MS Spectrum Peak List

m/z	Abundance
45738.85	5650.55
45811.51	6827.88
45848.62	8192.03
45878.16	8347.26
45904.18	6980.77
45944.76	5276.43
45962.38	4002.01
45986.05	5022.49
46015.74	4617.21
54103.93	3668.94

Supplementary Figure S1. Mass spectroscopy of **(a)** MrNV capsid protein and **(b)** folic acid (FA)-conjugated MrNV capsid protein. The calculated molecular mass for MrNV capsid protein is 45324.85 Da and the most abundant mass detected was 45333.03 Da. The calculated mass for one and two FA molecules conjugated to MrNV capsid protein is 45748.25 Da and 46171.66 Da, respectively. These values correspond well with the observed masses of 45738.85 Da and 46015.74 Da, respectively.

(a)

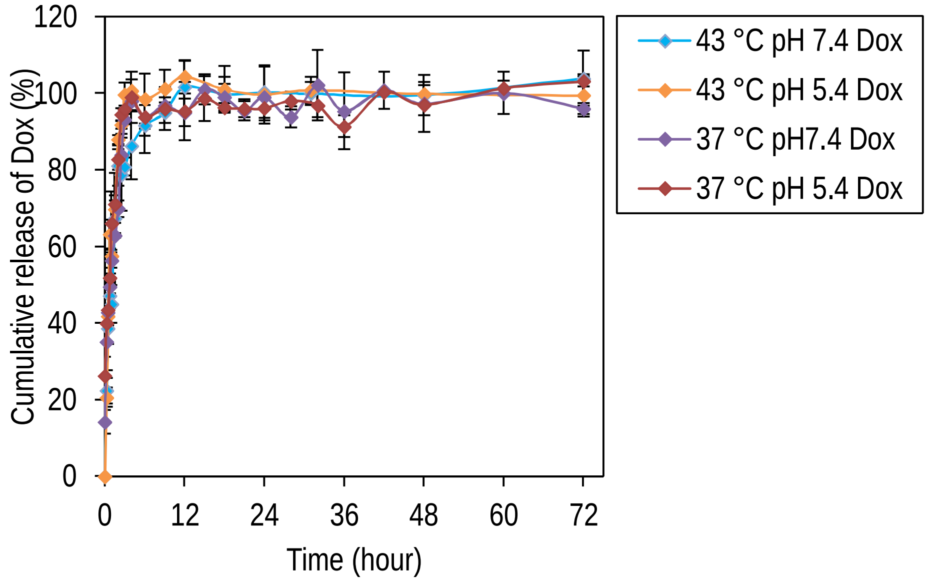

(b)

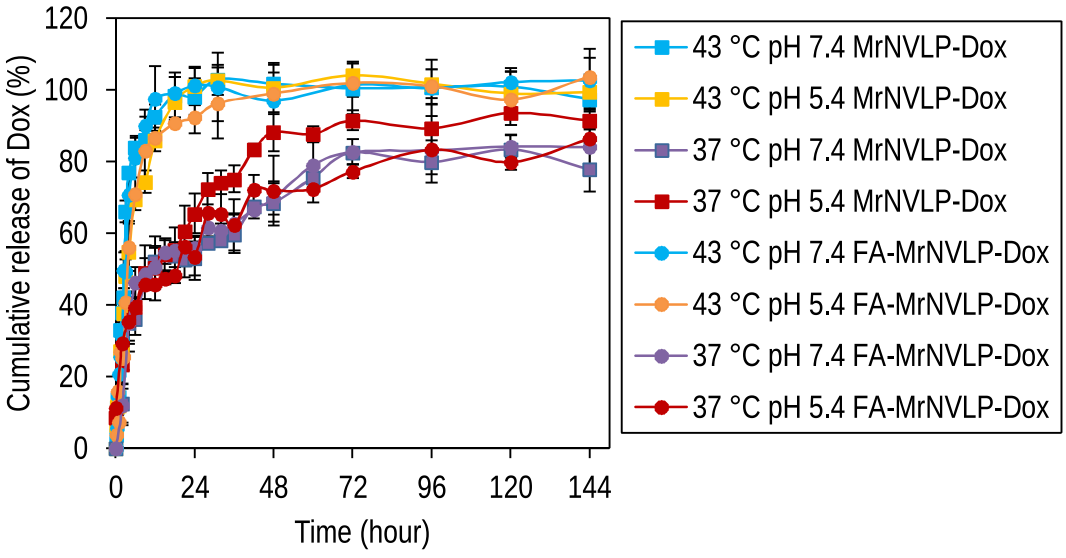

Supplementary Figure S2. Drug release profile of **(a)** free doxorubicin (Dox), and **(b)** virus-like particle of *Macrobrachium rosenbergii* nodavirus loaded with Dox (MrNVLP-Dox), and folic acid (FA)-conjugated-and-Dox-loaded MrNVLP (FA-MrNVLP-Dox) at different temperatures and pH. The drug release profile at pH 5.4 was not significantly different compared with that at pH 7.4. Instead, the Dox was released with a significantly higher rate at 43 °C as compared with that at 37 °C ($P<0.05$). These results indicate that the release of Dox from the nanoparticles was temperature dependent at pH 7.4 and 5.4.

(a)

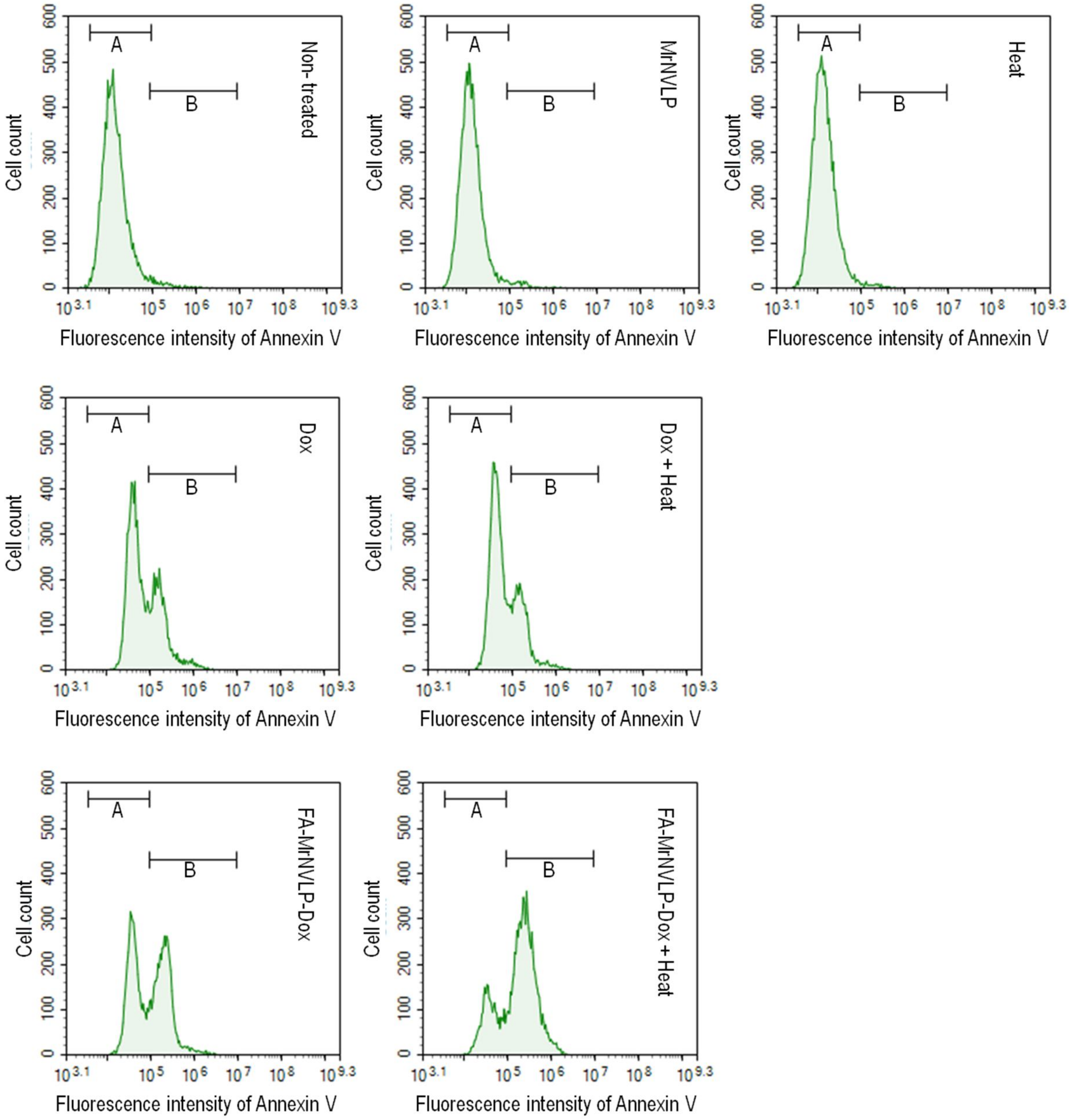
Non- treated
MrNVLP
Heat
Dox
Dox + Heat
FA-MrNVLP-Dox
FA-MrNVLP-Dox + Heat
A
B
Cell count
Fluorescence intensity of Annexin V

(b)

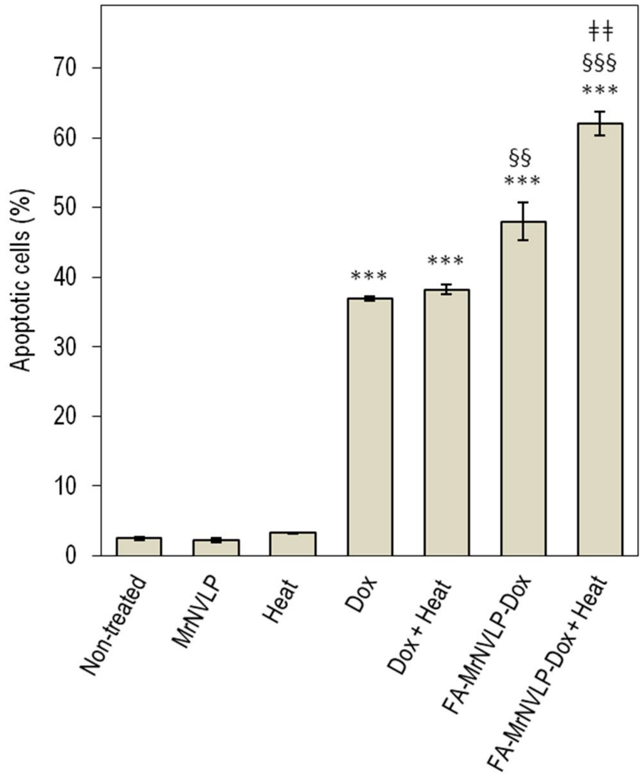

Apoptotic cells (%)
0
10
20
30
40
50
60
70
***
***
§§
***
ǂǂ
§§§
***
Non-treated
MrNVLP
Heat
Dox
Dox + Heat
FA-MrNVLP-Dox
FA-MrNVLP-Dox + Heat

(c)

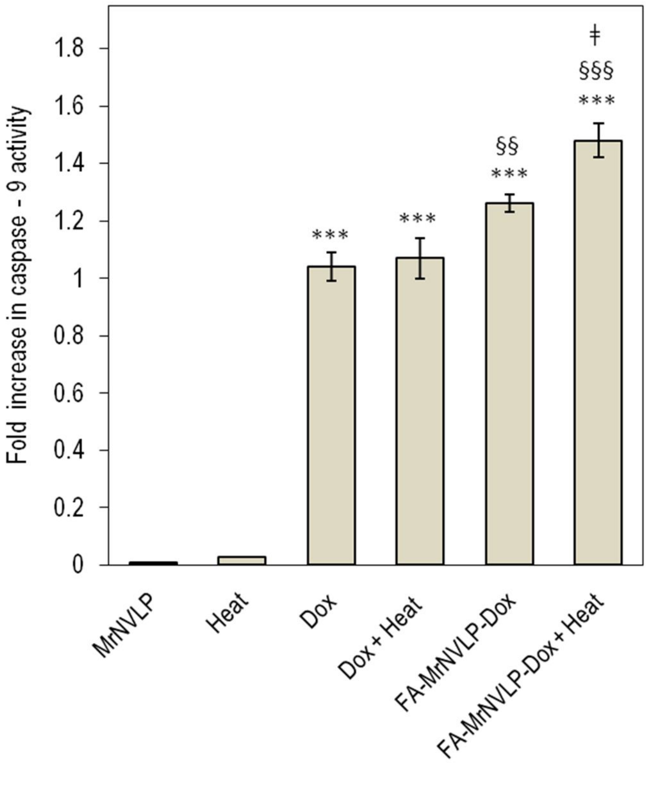

Fold increase in caspase - 9 activity
0
0.2
0.4
0.6
0.8
1
1.2
1.4
1.6
1.8
***
***
§§
***
ǂ
§§§
***
MrNVLP
Heat
Dox
Dox + Heat
FA-MrNVLP-Dox
FA-MrNVLP-Dox + Heat

Supplementary Figure S3. Apoptosis of HT29 cells. **(a)** Non-treated cells (Non-treated), cells incubated with virus-like particle of *Macrobrachium rosenbergii* nodavirus (MrNVLP), cells incubated at hyperthermia temperature (Heat), cells treated with free doxorubicin (Dox), Dox at hyperthermia temperature (Dox + Heat), folic acid (FA)-conjugated-and-Dox-loaded MrNVLP (FA-MrNVLP-Dox), and FA-MrNVLP-Dox at hyperthermia temperature (FA-MrNVLP-Dox + Heat) were stained with FITC annexin V and analysed by flow cytometry. Panels are representative of triplicate experiments. Apoptotic cells bound to FITC annexin V. 'A' represents healthy cells with low fluorescence intensity of FITC annexin V, while 'B' shows apoptotic cells shifted to higher fluorescence signals. **(b)** Percentage of apoptotic cells was calculated by comparing the ratio of cells in 'B' to the total amount of cells. **(c)** Caspase-9 assay of treated cells. Data are expressed as mean±standard deviation of triplicate experiments. *** indicates $P<0.001$ as compared with non-treated cells, §§§ indicates $P<0.001$ and §§ indicates $P<0.01$ as compared with cells treated with Dox, ǂǂ indicates $P<0.01$ and ǂ indicates $P<0.05$ when comparing between the cells treated with FA-MrNVLP-Dox with and without hyperthermia incubation.
